# Poly[[{μ_3_-tris­[2-(4-phenyl-1,2,3-triazol-1-yl)eth­yl]amine}silver(I)] hexa­fluorido­phosphate]

**DOI:** 10.1107/S1600536808028298

**Published:** 2008-09-13

**Authors:** Hiromi Ohi, Mayumi Shimizu, Makoto Obata, Takuzo Funabiki, Shigenobu Yano

**Affiliations:** a Endowed Research Section, Photomedical Science, Innovative Collaboration Center, Kyoto University, Kyoto-daigaku Katsura, Nishikyo-ku, Kyoto 615-8520, Japan; b Graduate School of Humanities and Sciences, Nara Women’s University, Kitauoyanishimachi, Nara 630-8506, Japan; c Department of Molecular Chemistry and Biochemistry, Factory of Science and Engineering, Doshisha University, Kyotanabe, Kyoto 610-0321, Japan

## Abstract

The title compound, {[Ag(*L*)]PF_6_)_*n*_ {*L* is tris­[2-(4-phenyl-1,2,3-triazol-1-yl)eth­yl]amine, C_30_H_30_N_10_}, consists of alternating two-dimensional cationic layers of [Ag(*L*)]^+^ and anionic PF_6_^−^ layers. Each Ag^I^ atom is three coordinated in a T-shaped geometry by three N atoms from three ligands. Each ligand links three Ag^I^ atoms, generating a two-dimensional network structure with two different metallacycles, *A* and *B*. In *A*, eight coordination units from four ligands connect four Ag^I^ atoms, forming a 48-membered ring. In *B*, four coordination units from two ligands link two Ag^I^ atoms, forming a 24-membered ring. Each *B* ring is surrounded by four *A* rings, and each *A* ring has four *A* and four *B* rings as neighbours. This cationic layer thus generates a 4.8^2^ topology network, with each Ag^I^ centre and ligand acting as a three-connected topological node.

## Related literature

For related literature, see: Newkome *et al.* (1999 ▶); Robin & Fromm (2006 ▶); Ohi *et al.* (2004 ▶, 2005 ▶); Obata *et al.* (2008 ▶).
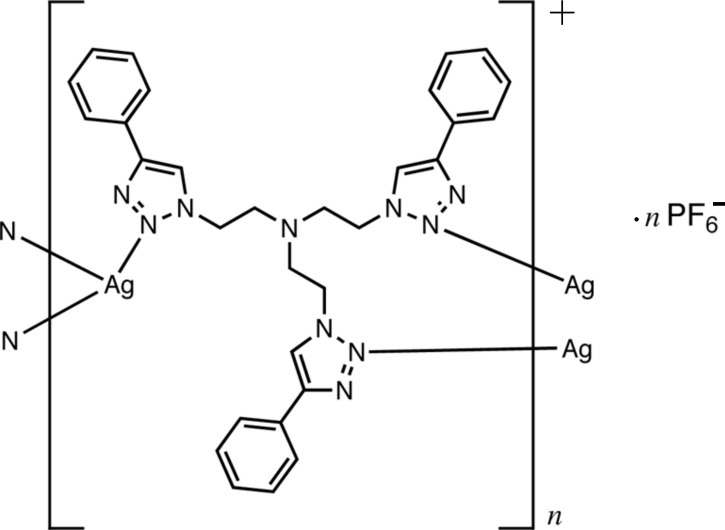


## Experimental

### 

#### Crystal data

[Ag(C_30_H_30_N_10_)]PF_6_
*M* *_r_* = 783.47
Monoclinic, 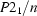

*a* = 14.893 (3) Å
*b* = 14.935 (3) Å
*c* = 15.735 (3) Å
β = 112.646 (5)°
*V* = 3230.2 (12) Å^3^
*Z* = 4
Mo *K*α radiation
μ = 0.75 mm^−1^
*T* = 193.1 K
0.30 × 0.15 × 0.05 mm

#### Data collection

Rigaku Mercury diffractometer
Absorption correction: multi-scan (Jacobson, 1998 ▶) *T*_min_ = 0.776, *T*_max_ = 0.963
31971 measured reflections
7326 independent reflections
4647 reflections with *F* ^2^ > 2σ(*F* ^2^)
*R*_int_ = 0.075

#### Refinement

*R*[*F* ^2^ > 2σ(*F* ^2^)] = 0.054
*wR*(*F* ^2^) = 0.055
*S* = 1.03
7326 reflections
463 parameters
All H-atom parameters refined
Δρ_max_ = 3.48 e Å^−3^
Δρ_min_ = −2.33 e Å^−3^

### 

Data collection: *CrystalClear* (Rigaku/MSC & Rigaku, 2006 ▶); cell refinement: *CrystalClear*; data reduction: *CrystalStructure*; program(s) used to solve structure: *SIR97* (Altomare *et al.*, 1999 ▶); program(s) used to refine structure: *CRYSTALS* (Betteridge *et al.*, 2003 ▶); molecular graphics: *ORTEPIII* (Burnett & Johnson, 1996 ▶); software used to prepare material for publication: *CrystalStructure* (Rigaku/MSC & Rigaku, 2006 ▶).

## Supplementary Material

Crystal structure: contains datablocks global, I. DOI: 10.1107/S1600536808028298/bt2780sup1.cif

Structure factors: contains datablocks I, I. DOI: 10.1107/S1600536808028298/bt2780Isup2.hkl

Additional supplementary materials:  crystallographic information; 3D view; checkCIF report

## Figures and Tables

**Table:** 

Ag1—N4	2.208 (2)
Ag1—N7^i^	2.210 (3)
Ag1—N10^ii^	2.268 (2)

**Table:** 

N4—Ag1—N7^i^	132.43 (10)
N4—Ag1—N10^ii^	114.02 (10)
N7^i^—Ag1—N10^ii^	113.51 (10)

Symmetry codes: (i) 
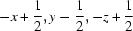
; (ii) 
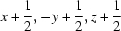
.

**Fig. 1.**
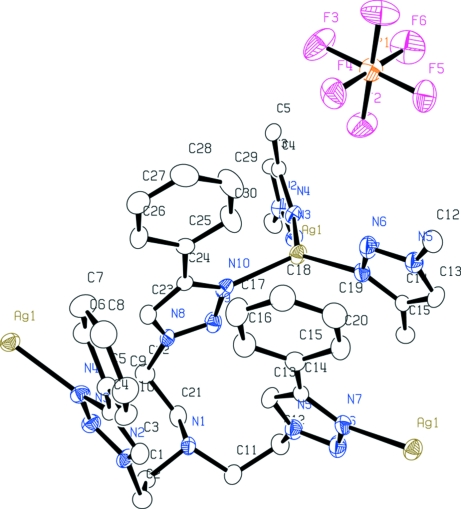
The coordination geometry of the AgI atom and the ligated mode of L in the title compound. Displacement ellipsoids are drawn at the 50% probability level. H atoms omitted for clarity.

**Fig. 2.**
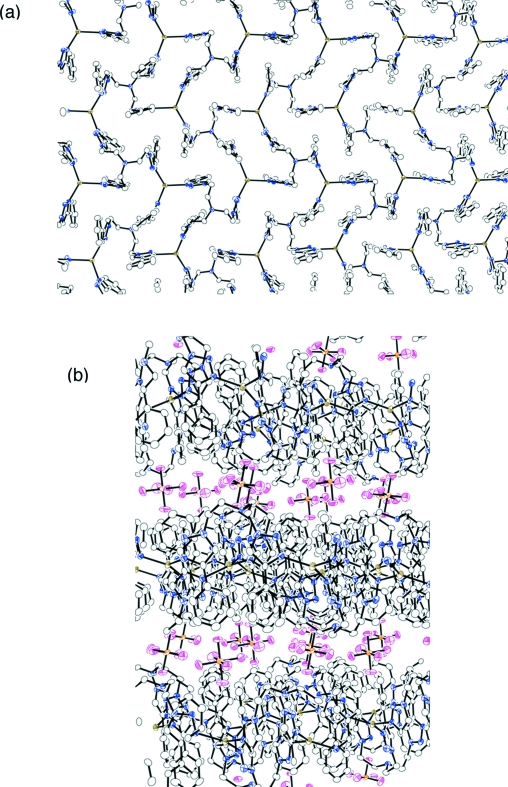
(a) Two-dimensional 4.82 topology network with 24-membered and 48-membered metallacycles. (b). Three-dimensional packing diagram, showing the alternate arrangement of cationic layers (black) and anionic layers (pink).

## supplementary crystallographic information

### Comment

Coordination polymer complexes have attracted much attention due to their various intriguing framework topologies and their unique properties such as magnetism, physical gas adsorption, ion-exchange, heterogeneous catalysis, and so on (Newkome *et al.*, 1999; Robin *et al.*, 2006). We have recently demonstrated that a *C*_3_ symmetric tripodal tripyridine ligand consisting of a 1,3,5-triethylbenzene spacer can be adopted in coordination polymer chemistry to give one- and/or two-dimensional coordination polymer complexes with a variety of network topology (Ohi *et al.*, 2004, 2005). In this study, we synthesized a new *C*_3_ symmetric lingad (*L*) consisting of triethylamine as the spacer and three 1,2,3-triazole groups as the metal binding site by using Huisgen reaction and used this ligand to synthesize a new Ag^I^ complex, [[Ag^I^(*L*)](PF_6_)]*_n_* (I). We report here the crystal structure of Ag^I^ complex.

In the title compound (I), the asymmetric unit contained one ligand molecular, one Ag^I^ ion, and one PF_6_^-^ counterion. No solvent molecules were incorporated in the structure. As shown in Fig. 1, each Ag^I^ ion features a T-shaped coordination geometry, being coordinated by three nitrogen atoms from three ligands [Ag—N: 2.208 (2)–2.268 (2) Å; N—Ag—N: 113.51 (10)–132.43 (10)°], and each ligand links three Ag^I^ atoms to generate a two-dimensional network structure with two different metallacycles A and B. In A, eight coordination moieties from four ligands connected four Ag^I^ atoms to form a 48-membered ring. In B, four coordination moieties from two ligands link two Ag^I^ atoms to form 24-membered ring. Each B ring is surrounded by four A rings, and each A ring neighbors upon four A and four B rings. Thus, this sheet generates a rare 4.8^2^ topology network with each Ag^I^ center and lingad acting as a three-connected topological node (Fig. 2a). The two-dimensional polymer sheets are stacked alternate arranging cationic two-dimensional layers of [Ag^I^(*L*)]^+^ and anionic (PF_6_^-^) layers to form laminated structure (Fig. 2b), where no specific interaction is probably between the sheets.

### Experimental

The ligand tris((4-phenyl-1,2,3-triazole-1-yl)ethyl)amine) (*L*) was synthesized by using Huisgen reaction, which is known as cycloaddition of azide and acetylene derivatives to give 1,2,3-triazole unit (Obata *et al.*, 2008). The title coordination complex, (I), was synthesized according to the following method. An acetone/CHCl_3_ (*v*/*v* = 1/1, 5 ml) solution of Ag^I^PF_6_ (50.6 mg, 2.0 *x* 10 ^-4^ mol) was added slowly to an acetone/CHCl_3_ (*v*/*v* = 1/1, 45 ml) solution of *L* (104.6 mg, 2.0 *x* 10 ^-4^ mol). After the mixture was stirred for 1 day under dark, the precipitate was collected by filtration to give white powder. This powder was dissolved in CH_3_CN and insoluble materials were removed by filtration. The filtrate was concentrated under reduced pressure to give white powder (98.6 mg, yield 64%). Single crystals suitable for X-ray crystallographic analysis were obtained by recrystallization from CH_3_CN/CHCl_3_ (*v*/*v* = 2/1)/Et_2_O. Anal. Calcd. for C_30_H_30_AgF_6_N_10_P: C 45.99, H 3.86, N 17.88. Found: C 46.17, H 4.00, N 17.80.

### Refinement

Hydrogen atoms were positioned geometrically (C—H = 0.95 Å) and refined using a riding model with U(H) = 1.2U_eq_(C).

### Crystal data

**Table:**

[Ag(C_30_H_30_N_10_)]PF_6_	*F*(000) = 1584.00
*M**_r_* = 783.47	*D*_x_ = 1.611 Mg m^−^^3^
Monoclinic, *P*2_1_/*n*	Mo *K*α radiation, λ = 0.71070 Å
Hall symbol: -P 2yn	Cell parameters from 9566 reflections
*a* = 14.893 (3) Å	θ = 4.0–27.5°
*b* = 14.935 (3) Å	µ = 0.75 mm^−^^1^
*c* = 15.735 (3) Å	*T* = 193 K
β = 112.646 (5)°	Platelet, colourless
*V* = 3230.2 (12) Å^3^	0.30 × 0.15 × 0.05 mm
*Z* = 4	

### Data collection

**Table:**

Rigaku Mercury diffractometer	4647 reflections with *F*^2^ > 2σ(*F*^2^)
Detector resolution: 7.31 pixels mm^-1^	*R*_int_ = 0.075
ω scans	θ_max_ = 27.5°
Absorption correction: multi-scan (Jacobson, 1998)	*h* = −19→16
*T*_min_ = 0.776, *T*_max_ = 0.963	*k* = −19→19
31971 measured reflections	*l* = −20→20
7326 independent reflections	

### Refinement

**Table:**

Refinement on *F*^2^	0 restraints
*R*[*F*^2^ > 2σ(*F*^2^)] = 0.054	All H-atom parameters refined
*wR*(*F*^2^) = 0.055	*w* = 1/[1.0000σ(*F*_o_^2^)]/(4*F*_o_^2^)
*S* = 1.03	(Δ/σ)_max_ < 0.001
7326 reflections	Δρ_max_ = 3.48 e Å^−^^3^
463 parameters	Δρ_min_ = −2.33 e Å^−^^3^

### Special details

**Table:**

Experimental. The ligand tris((4-phenyl-1,2,3-triazole-1-yl)ethyl)amine) (*L*) was synthesized by using Huisgen reaction, which is known as cycloaddition of azide and acetylene derivatives to give 1,2,3-triazole unit. The conversion of tris(2-chloroethyl)amine (2.21 g, 10.9 mmol) into tris(2-azidoethyl)amine was achieved by addition of 3 mole equivalents of sodium azide to tris(2-chloroethyl)amine in dimethylformamide under stirring at 80 °C. To the THF (100 ml) and water (100 ml) solution of crude tris(2-azidoethyl)amine obtained and phenylacethylene (2.57 g, 25.2 mmol) was added 1 *M* sodium ascorbate (aq) (0.9 ml) and 7.5 wt% CuSO_4_ (aq) (2.5 ml) and the mixture was stirred at 50 °C for 1 day. After concentration under reduced pressure, the resulting residue was then suspended in CHCl_3_, to which an aqueous solution of NH_3_ was successively added. After washing the organic layer with the NH_3_ aqueous solution, the organic layer was dried over anhydrous Na_2_SO_4_ and concentrated by evaporation. The resulting residue was purified by silica gel column chromatography (eluent; from CHCl_3_ to ethyl acetate). The organic materials having *R*_f_ = 0.21 (eluent; ethyl acetate) were collected (1.73 g, yield 37%). ^1^H NMR (CDCl_3_, 400 MHz): δ 3.33 (dt, 6H, *J* = 4.8, 2.4 Hz, N—CH_2_C*H*_2_–), 4.10 (dt, 6H, *J* = 4.8, 2.4 Hz, N—C*H*_2_CH_2_–), 6.80 (s, 3H, triazole-*H*), 7.00 (t, 6H, *J* = 7.6 Hz Ar-3*H*, 5*H*), 7.18 (tt, 3H, *J* = 7.6, 1.2 Hz, Ar-4*H*), 7.27 (d, 3H, *J* = 7.6 Hz, Ar-2*H* or 6*H*), 7.28 (d, 3H, *J* = 7.6 Hz, Ar-2*H* or 6*H*). HRMS (FAB, pos): *m/z* = 531.2729 calcd for [*L* + H]^+^, C_30_H_31_N_10_, 531.2733.
Geometry. ENTER SPECIAL DETAILS OF THE MOLECULAR GEOMETRY
Refinement. Refinement was performed using all reflections. The weighted *R*-factor (*wR*) and goodness of fit (*S*) are based on *F*^2^. *R*-factor (gt) are based on *F*. The threshold expression of *F*^2^ > 2.0 σ(*F*^2^) is used only for calculating *R*-factor (gt).

### Fractional atomic coordinates and isotropic or equivalent isotropic displacement parameters (Å^2^)

**Table:**

	*x*	*y*	*z*	*U*_iso_*/*U*_eq_	
Ag(1)	0.45614 (2)	0.23636 (2)	0.47433 (2)	0.02556 (7)	
P(1)	0.72081 (8)	0.21389 (8)	0.19893 (9)	0.0300 (3)	
F(1)	0.78327 (16)	0.23364 (19)	0.13875 (17)	0.0552 (8)	
F(2)	0.65821 (18)	0.19439 (16)	0.25993 (18)	0.0492 (9)	
F(3)	0.7415 (2)	0.31311 (17)	0.2382 (2)	0.0583 (10)	
F(4)	0.81644 (17)	0.17994 (18)	0.28073 (17)	0.0477 (8)	
F(5)	0.70048 (18)	0.11394 (16)	0.15986 (17)	0.0475 (8)	
F(6)	0.62526 (17)	0.2473 (2)	0.11681 (17)	0.0628 (9)	
N(1)	0.0406 (2)	0.4073 (2)	0.3528 (2)	0.0209 (9)	
N(2)	0.2601 (2)	0.4507 (2)	0.4393 (2)	0.0264 (10)	
N(3)	0.2869 (2)	0.3656 (2)	0.4548 (2)	0.0249 (10)	
N(4)	0.3631 (2)	0.3565 (2)	0.4315 (2)	0.0223 (9)	
N(5)	−0.0085 (2)	0.5197 (2)	0.1746 (2)	0.0258 (10)	
N(6)	−0.0277 (2)	0.5971 (2)	0.1282 (2)	0.0303 (10)	
N(7)	0.0412 (2)	0.6093 (2)	0.0966 (2)	0.0242 (9)	
N(8)	0.0859 (2)	0.2467 (2)	0.26377 (19)	0.0231 (9)	
N(9)	0.0191 (2)	0.2593 (2)	0.1787 (2)	0.0265 (9)	
N(10)	0.06704 (19)	0.2581 (2)	0.1214 (2)	0.0214 (8)	
C(1)	0.1011 (2)	0.4214 (2)	0.4501 (2)	0.0245 (11)	
C(2)	0.1823 (3)	0.4872 (2)	0.4670 (3)	0.0290 (13)	
C(3)	0.3162 (3)	0.4970 (2)	0.4061 (2)	0.0258 (12)	
C(4)	0.3837 (2)	0.4368 (2)	0.4008 (2)	0.0196 (10)	
C(5)	0.4644 (2)	0.4490 (2)	0.3710 (2)	0.0216 (11)	
C(6)	0.5077 (3)	0.3770 (2)	0.3441 (3)	0.0320 (13)	
C(7)	0.5821 (3)	0.3898 (2)	0.3148 (3)	0.0355 (13)	
C(8)	0.6151 (3)	0.4762 (2)	0.3095 (3)	0.0339 (13)	
C(9)	0.5733 (3)	0.5481 (2)	0.3355 (3)	0.0334 (13)	
C(10)	0.4994 (2)	0.5343 (2)	0.3659 (2)	0.0262 (12)	
C(11)	−0.0307 (2)	0.4794 (2)	0.3191 (2)	0.0275 (12)	
C(12)	−0.0760 (3)	0.4862 (2)	0.2144 (2)	0.0292 (12)	
C(13)	0.0726 (3)	0.4829 (2)	0.1727 (2)	0.0289 (12)	
C(14)	0.1058 (2)	0.5395 (2)	0.1229 (2)	0.0213 (11)	
C(15)	0.1908 (3)	0.5339 (2)	0.0964 (3)	0.0260 (12)	
C(16)	0.2701 (3)	0.4828 (2)	0.1481 (3)	0.0338 (13)	
C(17)	0.3512 (3)	0.4771 (3)	0.1237 (3)	0.0435 (15)	
C(18)	0.3528 (3)	0.5245 (3)	0.0501 (3)	0.0378 (14)	
C(19)	0.2756 (3)	0.5757 (2)	−0.0014 (3)	0.0373 (14)	
C(20)	0.1933 (3)	0.5808 (2)	0.0213 (3)	0.0330 (13)	
C(21)	−0.0095 (2)	0.3209 (2)	0.3415 (2)	0.0264 (12)	
C(22)	0.0557 (2)	0.2428 (2)	0.3421 (2)	0.0305 (11)	
C(23)	0.1756 (2)	0.2375 (2)	0.2625 (2)	0.0246 (10)	
C(24)	0.1642 (2)	0.2460 (2)	0.1730 (2)	0.0186 (9)	
C(25)	0.2370 (2)	0.2422 (2)	0.1313 (2)	0.0229 (10)	
C(26)	0.3333 (2)	0.2192 (2)	0.1856 (2)	0.0300 (12)	
C(27)	0.4044 (2)	0.2207 (2)	0.1484 (2)	0.0348 (13)	
C(28)	0.3802 (2)	0.2411 (3)	0.0577 (3)	0.0398 (13)	
C(29)	0.2856 (3)	0.2615 (3)	0.0021 (2)	0.0463 (14)	
C(30)	0.2140 (2)	0.2632 (3)	0.0378 (2)	0.0371 (12)	
H(1)	0.0609	0.4411	0.4809	0.031*	
H(2)	0.1300	0.3657	0.4752	0.031*	
H(3)	0.2097	0.5019	0.5306	0.035*	
H(4)	0.1560	0.5396	0.4320	0.035*	
H(5)	0.3109	0.5585	0.3894	0.030*	
H(6)	0.4843	0.3180	0.3456	0.038*	
H(7)	0.6119	0.3401	0.2984	0.043*	
H(8)	0.6660	0.4853	0.2882	0.042*	
H(9)	0.5956	0.6070	0.3318	0.039*	
H(10)	0.4713	0.5841	0.3842	0.032*	
H(11)	−0.0812	0.4703	0.3410	0.033*	
H(12)	0.0015	0.5343	0.3426	0.033*	
H(13)	−0.1302	0.5256	0.1973	0.034*	
H(14)	−0.0975	0.4283	0.1902	0.035*	
H(15)	0.1018	0.4283	0.2008	0.034*	
H(16)	0.2699	0.4520	0.2009	0.042*	
H(17)	0.4051	0.4408	0.1587	0.051*	
H(18)	0.4077	0.5204	0.0335	0.046*	
H(19)	0.2777	0.6090	−0.0521	0.046*	
H(20)	0.1390	0.6162	−0.0148	0.038*	
H(21)	−0.0344	0.3136	0.3883	0.033*	
H(22)	−0.0618	0.3215	0.2829	0.033*	
H(23)	0.0215	0.1885	0.3391	0.038*	
H(24)	0.1117	0.2447	0.3979	0.038*	
H(25)	0.2345	0.2270	0.3140	0.029*	
H(26)	0.3498	0.2023	0.2480	0.033*	
H(27)	0.4700	0.2075	0.1866	0.041*	
H(28)	0.4292	0.2411	0.0330	0.049*	
H(29)	0.2695	0.2747	−0.0612	0.056*	
H(30)	0.1488	0.2782	−0.0003	0.043*	

### Atomic displacement parameters (Å^2^)

**Table:**

	*U*^11^	*U*^22^	*U*^33^	*U*^12^	*U*^13^	*U*^23^
Ag(1)	0.02591 (16)	0.01924 (15)	0.02906 (17)	0.00118 (17)	0.00786 (13)	−0.00264 (17)
P(1)	0.0251 (6)	0.0333 (7)	0.0370 (7)	0.0018 (5)	0.0179 (5)	−0.0044 (5)
F(1)	0.0571 (16)	0.0648 (18)	0.0679 (18)	0.0167 (16)	0.0508 (15)	0.0146 (17)
F(2)	0.0509 (16)	0.0533 (17)	0.066 (2)	−0.0082 (13)	0.0474 (15)	−0.0081 (14)
F(3)	0.059 (2)	0.0343 (16)	0.100 (2)	−0.0089 (14)	0.0517 (19)	−0.0210 (15)
F(4)	0.0291 (15)	0.0657 (19)	0.0396 (16)	−0.0025 (13)	0.0036 (13)	−0.0018 (13)
F(5)	0.0530 (17)	0.0392 (16)	0.0500 (17)	−0.0004 (13)	0.0195 (14)	−0.0172 (13)
F(6)	0.0385 (14)	0.073 (2)	0.0681 (18)	0.0214 (17)	0.0110 (13)	0.0161 (18)
N(1)	0.0190 (18)	0.0246 (19)	0.0191 (18)	0.0041 (14)	0.0073 (15)	0.0046 (14)
N(2)	0.0191 (19)	0.027 (2)	0.032 (2)	0.0078 (15)	0.0085 (17)	−0.0015 (16)
N(3)	0.025 (2)	0.0202 (19)	0.028 (2)	0.0007 (15)	0.0092 (17)	−0.0004 (15)
N(4)	0.0207 (18)	0.0194 (18)	0.027 (2)	−0.0013 (14)	0.0091 (16)	−0.0030 (15)
N(5)	0.027 (2)	0.024 (2)	0.024 (2)	−0.0012 (16)	0.0073 (17)	0.0025 (16)
N(6)	0.033 (2)	0.028 (2)	0.027 (2)	0.0027 (17)	0.0082 (18)	0.0069 (16)
N(7)	0.029 (2)	0.0230 (19)	0.0200 (19)	0.0010 (15)	0.0080 (16)	0.0034 (15)
N(8)	0.0319 (18)	0.0146 (19)	0.0206 (17)	0.0029 (15)	0.0076 (15)	0.0042 (15)
N(9)	0.0206 (16)	0.0262 (19)	0.0275 (18)	−0.0016 (16)	0.0035 (14)	0.0025 (17)
N(10)	0.0153 (15)	0.0141 (17)	0.0324 (18)	−0.0031 (14)	0.0065 (14)	−0.0005 (15)
C(1)	0.023 (2)	0.036 (2)	0.019 (2)	0.0077 (19)	0.0132 (19)	0.0000 (19)
C(2)	0.026 (2)	0.037 (2)	0.026 (2)	0.009 (2)	0.012 (2)	−0.002 (2)
C(3)	0.029 (2)	0.021 (2)	0.025 (2)	−0.0017 (18)	0.008 (2)	0.0042 (18)
C(4)	0.020 (2)	0.016 (2)	0.019 (2)	0.0022 (16)	0.0027 (18)	0.0006 (16)
C(5)	0.022 (2)	0.017 (2)	0.024 (2)	0.0005 (17)	0.0068 (19)	−0.0013 (17)
C(6)	0.033 (2)	0.023 (2)	0.039 (2)	−0.0044 (19)	0.012 (2)	−0.004 (2)
C(7)	0.028 (2)	0.034 (2)	0.045 (3)	0.003 (2)	0.015 (2)	−0.006 (2)
C(8)	0.024 (2)	0.041 (2)	0.040 (2)	−0.004 (2)	0.016 (2)	0.001 (2)
C(9)	0.028 (2)	0.026 (2)	0.044 (2)	−0.007 (2)	0.011 (2)	−0.002 (2)
C(10)	0.026 (2)	0.022 (2)	0.033 (2)	0.0018 (18)	0.013 (2)	0.0001 (19)
C(11)	0.023 (2)	0.030 (2)	0.029 (2)	0.0057 (19)	0.0093 (19)	0.0033 (19)
C(12)	0.026 (2)	0.034 (2)	0.026 (2)	0.0013 (19)	0.009 (2)	0.003 (2)
C(13)	0.034 (2)	0.020 (2)	0.031 (2)	0.0055 (19)	0.010 (2)	0.0060 (19)
C(14)	0.028 (2)	0.016 (2)	0.016 (2)	−0.0004 (18)	0.0034 (19)	−0.0009 (16)
C(15)	0.030 (2)	0.020 (2)	0.025 (2)	−0.0051 (19)	0.006 (2)	−0.0044 (18)
C(16)	0.037 (2)	0.032 (2)	0.035 (2)	−0.002 (2)	0.018 (2)	0.007 (2)
C(17)	0.037 (2)	0.048 (3)	0.042 (3)	0.010 (2)	0.012 (2)	0.009 (2)
C(18)	0.029 (2)	0.046 (3)	0.040 (2)	0.002 (2)	0.014 (2)	−0.000 (2)
C(19)	0.049 (3)	0.031 (2)	0.036 (2)	−0.006 (2)	0.020 (2)	0.003 (2)
C(20)	0.040 (2)	0.026 (2)	0.029 (2)	0.000 (2)	0.009 (2)	0.006 (2)
C(21)	0.025 (2)	0.031 (2)	0.026 (2)	0.0000 (19)	0.0136 (19)	0.0095 (19)
C(22)	0.041 (2)	0.027 (2)	0.028 (2)	−0.001 (2)	0.018 (2)	0.004 (2)
C(23)	0.020 (2)	0.029 (2)	0.024 (2)	0.004 (2)	0.0074 (17)	0.003 (2)
C(24)	0.0205 (19)	0.009 (2)	0.0212 (19)	−0.0041 (16)	0.0022 (16)	−0.0047 (16)
C(25)	0.0212 (19)	0.021 (2)	0.023 (2)	−0.0004 (18)	0.0052 (16)	−0.0066 (19)
C(26)	0.026 (2)	0.026 (2)	0.030 (2)	0.0029 (19)	0.003 (2)	0.0026 (19)
C(27)	0.020 (2)	0.041 (2)	0.040 (2)	0.005 (2)	0.008 (2)	−0.005 (2)
C(28)	0.030 (2)	0.051 (3)	0.042 (2)	−0.004 (2)	0.019 (2)	−0.011 (2)
C(29)	0.039 (2)	0.072 (3)	0.029 (2)	−0.006 (2)	0.014 (2)	0.002 (2)
C(30)	0.025 (2)	0.058 (2)	0.024 (2)	−0.005 (2)	0.0050 (18)	0.003 (2)

### Geometric parameters (Å, °)

**Table:**

Ag(1)—N(4)	2.208 (2)	C(17)—C(18)	1.365 (7)
Ag(1)—N(7)^i^	2.210 (3)	C(18)—C(19)	1.359 (5)
Ag(1)—N(10)^ii^	2.268 (2)	C(19)—C(20)	1.405 (8)
P(1)—F(1)	1.590 (3)	C(21)—C(22)	1.516 (5)
P(1)—F(2)	1.601 (3)	C(23)—C(24)	1.359 (5)
P(1)—F(3)	1.590 (2)	C(24)—C(25)	1.468 (6)
P(1)—F(4)	1.591 (2)	C(25)—C(26)	1.401 (4)
P(1)—F(5)	1.598 (2)	C(25)—C(30)	1.410 (5)
P(1)—F(6)	1.590 (2)	C(26)—C(27)	1.395 (7)
N(1)—C(1)	1.462 (4)	C(27)—C(28)	1.365 (6)
N(1)—C(11)	1.462 (4)	C(28)—C(29)	1.376 (5)
N(1)—C(21)	1.466 (4)	C(29)—C(30)	1.385 (7)
N(2)—N(3)	1.327 (4)	C(1)—H(1)	0.950
N(2)—C(2)	1.489 (6)	C(1)—H(2)	0.950
N(2)—C(3)	1.336 (6)	C(2)—H(3)	0.950
N(3)—N(4)	1.327 (5)	C(2)—H(4)	0.950
N(4)—C(4)	1.372 (5)	C(3)—H(5)	0.950
N(5)—N(6)	1.338 (4)	C(6)—H(6)	0.950
N(5)—C(12)	1.463 (6)	C(7)—H(7)	0.950
N(5)—C(13)	1.337 (6)	C(8)—H(8)	0.950
N(6)—N(7)	1.314 (6)	C(9)—H(9)	0.950
N(7)—C(14)	1.370 (4)	C(10)—H(10)	0.950
N(8)—N(9)	1.339 (3)	C(11)—H(11)	0.950
N(8)—C(22)	1.466 (5)	C(11)—H(12)	0.950
N(8)—C(23)	1.350 (5)	C(12)—H(13)	0.950
N(9)—N(10)	1.349 (5)	C(12)—H(14)	0.950
N(10)—C(24)	1.372 (3)	C(13)—H(15)	0.950
C(1)—C(2)	1.501 (5)	C(16)—H(16)	0.950
C(3)—C(4)	1.375 (6)	C(17)—H(17)	0.950
C(4)—C(5)	1.460 (6)	C(18)—H(18)	0.950
C(5)—C(6)	1.402 (6)	C(19)—H(19)	0.950
C(5)—C(10)	1.390 (5)	C(20)—H(20)	0.950
C(6)—C(7)	1.368 (7)	C(21)—H(21)	0.950
C(7)—C(8)	1.394 (6)	C(21)—H(22)	0.950
C(8)—C(9)	1.381 (6)	C(22)—H(23)	0.950
C(9)—C(10)	1.374 (7)	C(22)—H(24)	0.950
C(11)—C(12)	1.524 (5)	C(23)—H(25)	0.950
C(13)—C(14)	1.368 (6)	C(26)—H(26)	0.950
C(14)—C(15)	1.479 (7)	C(27)—H(27)	0.950
C(15)—C(16)	1.379 (5)	C(28)—H(28)	0.950
C(15)—C(20)	1.386 (6)	C(29)—H(29)	0.950
C(16)—C(17)	1.404 (8)	C(30)—H(30)	0.950
			
F(1)···N(3)^iii^	3.271 (4)	N(10)···H(20)^ix^	3.433
F(1)···N(9)^iv^	3.343 (3)	C(4)···H(10)^vii^	3.253
F(1)···C(9)^v^	3.427 (5)	C(7)···H(3)^vii^	3.513
F(1)···C(19)^vi^	3.477 (5)	C(8)···H(3)^vii^	2.863
F(2)···N(6)^i^	3.409 (5)	C(9)···H(3)^vii^	3.195
F(2)···C(7)	3.362 (5)	C(11)···H(1)^xiii^	3.550
F(2)···C(12)^i^	3.421 (5)	C(12)···H(19)^ix^	3.414
F(2)···C(27)	3.523 (4)	C(12)···H(20)^ix^	3.289
F(3)···C(7)	3.256 (6)	C(13)···H(20)^ix^	3.502
F(3)···C(8)	3.516 (5)	C(19)···H(13)^ix^	3.368
F(3)···C(21)^iv^	3.430 (4)	C(19)···H(14)^ix^	3.131
F(4)···C(9)^v^	3.498 (5)	C(20)···H(13)^ix^	3.572
F(4)···C(21)^iv^	3.188 (4)	C(20)···H(14)^ix^	3.079
F(4)···C(22)^iv^	3.445 (4)	C(21)···H(28)^viii^	3.591
F(5)···C(1)^iii^	3.099 (4)	C(28)···H(21)^iii^	3.465
F(5)···C(2)^iii^	3.307 (5)	H(1)···F(5)^viii^	2.901
F(5)···C(8)^v^	3.316 (5)	H(1)···F(6)^viii^	3.440
F(5)···C(9)^v^	3.484 (5)	H(1)···C(11)^xiii^	3.550
F(5)···C(11)^i^	3.345 (5)	H(1)···H(1)^xiii^	2.754
F(6)···C(1)^iii^	3.554 (4)	H(1)···H(11)^xiii^	3.006
F(6)···C(27)	3.533 (5)	H(1)···H(12)^xiii^	3.271
F(6)···C(28)	3.403 (4)	H(2)···P(1)^viii^	3.460
N(2)···C(9)^vii^	3.456 (4)	H(2)···F(1)^viii^	3.084
N(3)···F(1)^viii^	3.271 (4)	H(2)···F(5)^viii^	2.700
N(3)···C(9)^vii^	3.405 (4)	H(2)···F(6)^viii^	2.818
N(4)···C(10)^vii^	3.463 (4)	H(3)···F(5)^viii^	2.714
N(5)···C(20)^ix^	3.578 (4)	H(3)···C(7)^vii^	3.513
N(6)···F(2)^x^	3.409 (5)	H(3)···C(8)^vii^	2.863
N(9)···F(1)^xi^	3.343 (3)	H(3)···C(9)^vii^	3.195
C(1)···F(5)^viii^	3.099 (4)	H(3)···H(8)^vii^	2.744
C(1)···F(6)^viii^	3.554 (4)	H(3)···H(9)^vii^	3.303
C(2)···F(5)^viii^	3.307 (5)	H(3)···H(11)^xiii^	3.300
C(4)···C(10)^vii^	3.431 (5)	H(7)···P(1)	3.254
C(7)···F(2)	3.362 (5)	H(7)···F(2)	2.429
C(7)···F(3)	3.256 (6)	H(7)···F(3)	2.489
C(8)···F(3)	3.516 (5)	H(7)···F(6)	3.251
C(8)···F(5)^xii^	3.316 (5)	H(8)···F(3)	3.030
C(9)···F(1)^xii^	3.427 (5)	H(8)···F(4)^xii^	3.148
C(9)···F(4)^xii^	3.498 (5)	H(8)···F(5)^xii^	2.657
C(9)···F(5)^xii^	3.484 (5)	H(8)···H(3)^vii^	2.744
C(9)···N(2)^vii^	3.456 (4)	H(8)···H(11)^iv^	3.536
C(9)···N(3)^vii^	3.405 (4)	H(9)···P(1)^xii^	3.358
C(10)···N(4)^vii^	3.463 (4)	H(9)···F(1)^xii^	2.530
C(10)···C(4)^vii^	3.431 (5)	H(9)···F(4)^xii^	2.797
C(11)···F(5)^x^	3.345 (5)	H(9)···F(5)^xii^	2.990
C(12)···F(2)^x^	3.421 (5)	H(9)···N(2)^vii^	3.517
C(12)···C(20)^ix^	3.582 (5)	H(9)···N(3)^vii^	3.159
C(19)···F(1)^vi^	3.477 (5)	H(9)···N(4)^vii^	3.576
C(20)···N(5)^ix^	3.578 (4)	H(9)···H(3)^vii^	3.303
C(20)···C(12)^ix^	3.582 (5)	H(10)···Ag(1)^vii^	3.386
C(21)···F(3)^xi^	3.430 (4)	H(10)···N(3)^vii^	3.595
C(21)···F(4)^xi^	3.188 (4)	H(10)···N(4)^vii^	3.127
C(22)···F(4)^xi^	3.445 (4)	H(10)···C(4)^vii^	3.253
C(27)···F(2)	3.523 (4)	H(11)···F(3)^xi^	3.437
C(27)···F(6)	3.533 (5)	H(11)···F(5)^x^	2.782
C(28)···F(6)	3.403 (4)	H(11)···H(1)^xiii^	3.006
Ag(1)···H(10)^vii^	3.386	H(11)···H(3)^xiii^	3.300
P(1)···H(2)^iii^	3.460	H(11)···H(8)^xi^	3.536
P(1)···H(7)	3.254	H(12)···F(2)^x^	3.320
P(1)···H(9)^v^	3.358	H(12)···F(5)^x^	3.221
P(1)···H(19)^vi^	3.519	H(12)···H(1)^xiii^	3.271
P(1)···H(22)^iv^	3.394	H(13)···F(2)^x^	2.682
P(1)···H(29)^ii^	3.564	H(13)···F(5)^x^	3.114
F(1)···H(2)^iii^	3.084	H(13)···C(19)^ix^	3.368
F(1)···H(9)^v^	2.530	H(13)···C(20)^ix^	3.572
F(1)···H(14)^iv^	3.340	H(13)···H(19)^ix^	3.198
F(1)···H(19)^vi^	2.694	H(13)···H(20)^ix^	3.530
F(1)···H(20)^vi^	3.451	H(14)···F(1)^xi^	3.340
F(1)···H(22)^iv^	2.857	H(14)···F(3)^xi^	3.267
F(2)···H(7)	2.429	H(14)···C(19)^ix^	3.131
F(2)···H(12)^i^	3.320	H(14)···C(20)^ix^	3.079
F(2)···H(13)^i^	2.682	H(14)···H(19)^ix^	2.783
F(2)···H(27)	2.595	H(14)···H(20)^ix^	2.670
F(2)···H(29)^ii^	2.700	H(18)···H(18)^vi^	3.360
F(3)···H(7)	2.489	H(19)···P(1)^vi^	3.519
F(3)···H(8)	3.030	H(19)···F(1)^vi^	2.694
F(3)···H(11)^iv^	3.437	H(19)···F(3)^vi^	3.062
F(3)···H(14)^iv^	3.267	H(19)···F(6)^vi^	2.978
F(3)···H(19)^vi^	3.062	H(19)···C(12)^ix^	3.414
F(3)···H(21)^iv^	3.263	H(19)···H(13)^ix^	3.198
F(3)···H(22)^iv^	2.740	H(19)···H(14)^ix^	2.783
F(3)···H(29)^ii^	3.293	H(20)···F(1)^vi^	3.451
F(4)···H(8)^v^	3.148	H(20)···N(5)^ix^	3.234
F(4)···H(9)^v^	2.797	H(20)···N(9)^ix^	3.312
F(4)···H(21)^iv^	2.981	H(20)···N(10)^ix^	3.433
F(4)···H(22)^iv^	2.777	H(20)···C(12)^ix^	3.289
F(4)···H(23)^iv^	2.833	H(20)···C(13)^ix^	3.502
F(4)···H(29)^ii^	2.912	H(20)···H(13)^ix^	3.530
F(5)···H(1)^iii^	2.901	H(20)···H(14)^ix^	2.670
F(5)···H(2)^iii^	2.700	H(21)···F(3)^xi^	3.263
F(5)···H(3)^iii^	2.714	H(21)···F(4)^xi^	2.981
F(5)···H(8)^v^	2.657	H(21)···F(6)^viii^	3.582
F(5)···H(9)^v^	2.990	H(21)···C(28)^viii^	3.465
F(5)···H(11)^i^	2.782	H(21)···H(28)^viii^	2.664
F(5)···H(12)^i^	3.221	H(21)···H(29)^viii^	3.563
F(5)···H(13)^i^	3.114	H(22)···P(1)^xi^	3.394
F(6)···H(1)^iii^	3.440	H(22)···F(1)^xi^	2.857
F(6)···H(2)^iii^	2.818	H(22)···F(3)^xi^	2.740
F(6)···H(7)	3.251	H(22)···F(4)^xi^	2.777
F(6)···H(19)^vi^	2.978	H(23)···F(4)^xi^	2.833
F(6)···H(21)^iii^	3.582	H(24)···F(6)^viii^	3.374
F(6)···H(24)^iii^	3.374	H(27)···F(2)	2.595
F(6)···H(27)	2.977	H(27)···F(6)	2.977
F(6)···H(28)	2.703	H(28)···F(6)	2.703
N(2)···H(9)^vii^	3.517	H(28)···C(21)^iii^	3.591
N(3)···H(9)^vii^	3.159	H(28)···H(21)^iii^	2.664
N(3)···H(10)^vii^	3.595	H(29)···P(1)^xiv^	3.564
N(4)···H(9)^vii^	3.576	H(29)···F(2)^xiv^	2.700
N(4)···H(10)^vii^	3.127	H(29)···F(3)^xiv^	3.293
N(5)···H(20)^ix^	3.234	H(29)···F(4)^xiv^	2.912
N(6)···H(30)^ix^	2.825	H(29)···H(21)^iii^	3.563
N(7)···H(30)^ix^	3.136	H(30)···N(6)^ix^	2.825
N(9)···H(20)^ix^	3.312	H(30)···N(7)^ix^	3.136
			
N(4)—Ag(1)—N(7)^i^	132.43 (10)	C(23)—C(24)—C(25)	129.9 (2)
N(4)—Ag(1)—N(10)^ii^	114.02 (10)	C(24)—C(25)—C(26)	119.7 (3)
N(7)^i^—Ag(1)—N(10)^ii^	113.51 (10)	C(24)—C(25)—C(30)	122.2 (3)
F(1)—P(1)—F(2)	179.70 (15)	C(26)—C(25)—C(30)	118.1 (4)
F(1)—P(1)—F(3)	89.34 (18)	C(25)—C(26)—C(27)	120.3 (3)
F(1)—P(1)—F(4)	89.67 (14)	C(26)—C(27)—C(28)	120.3 (3)
F(1)—P(1)—F(5)	90.68 (16)	C(27)—C(28)—C(29)	120.6 (4)
F(1)—P(1)—F(6)	90.20 (15)	C(28)—C(29)—C(30)	120.4 (4)
F(2)—P(1)—F(3)	90.39 (17)	C(25)—C(30)—C(29)	120.2 (3)
F(2)—P(1)—F(4)	90.20 (15)	N(1)—C(1)—H(1)	108.9
F(2)—P(1)—F(5)	89.59 (15)	N(1)—C(1)—H(2)	107.9
F(2)—P(1)—F(6)	89.93 (14)	C(2)—C(1)—H(1)	109.2
F(3)—P(1)—F(4)	90.18 (14)	C(2)—C(1)—H(2)	107.1
F(3)—P(1)—F(5)	179.69 (15)	H(1)—C(1)—H(2)	109.5
F(3)—P(1)—F(6)	90.13 (15)	N(2)—C(2)—H(3)	109.0
F(4)—P(1)—F(5)	89.51 (13)	N(2)—C(2)—H(4)	109.0
F(4)—P(1)—F(6)	179.66 (16)	C(1)—C(2)—H(3)	109.2
F(5)—P(1)—F(6)	90.17 (14)	C(1)—C(2)—H(4)	108.3
C(1)—N(1)—C(11)	110.0 (3)	H(3)—C(2)—H(4)	109.5
C(1)—N(1)—C(21)	109.5 (3)	N(2)—C(3)—H(5)	127.7
C(11)—N(1)—C(21)	109.9 (2)	C(4)—C(3)—H(5)	126.8
N(3)—N(2)—C(2)	120.7 (3)	C(5)—C(6)—H(6)	119.2
N(3)—N(2)—C(3)	112.2 (3)	C(7)—C(6)—H(6)	119.4
C(2)—N(2)—C(3)	126.8 (3)	C(6)—C(7)—H(7)	120.3
N(2)—N(3)—N(4)	106.0 (3)	C(8)—C(7)—H(7)	119.7
Ag(1)—N(4)—N(3)	120.1 (2)	C(7)—C(8)—H(8)	120.1
Ag(1)—N(4)—C(4)	128.3 (2)	C(9)—C(8)—H(8)	120.4
N(3)—N(4)—C(4)	109.8 (3)	C(8)—C(9)—H(9)	119.5
N(6)—N(5)—C(12)	119.4 (3)	C(10)—C(9)—H(9)	120.5
N(6)—N(5)—C(13)	110.4 (3)	C(5)—C(10)—H(10)	118.8
C(12)—N(5)—C(13)	130.1 (3)	C(9)—C(10)—H(10)	119.5
N(5)—N(6)—N(7)	106.9 (3)	N(1)—C(11)—H(11)	109.2
Ag(1)^x^—N(7)—N(6)	118.6 (2)	N(1)—C(11)—H(12)	107.9
Ag(1)^x^—N(7)—C(14)	131.2 (3)	C(12)—C(11)—H(11)	108.6
N(6)—N(7)—C(14)	110.0 (3)	C(12)—C(11)—H(12)	108.0
N(9)—N(8)—C(22)	119.7 (3)	H(11)—C(11)—H(12)	109.5
N(9)—N(8)—C(23)	111.0 (3)	N(5)—C(12)—H(13)	108.4
C(22)—N(8)—C(23)	129.3 (2)	N(5)—C(12)—H(14)	108.7
N(8)—N(9)—N(10)	106.6 (2)	C(11)—C(12)—H(13)	108.8
Ag(1)^xiv^—N(10)—N(9)	108.43 (17)	C(11)—C(12)—H(14)	108.2
Ag(1)^xiv^—N(10)—C(24)	142.8 (2)	H(13)—C(12)—H(14)	109.5
N(9)—N(10)—C(24)	108.4 (2)	N(5)—C(13)—H(15)	126.8
N(1)—C(1)—C(2)	114.2 (3)	C(14)—C(13)—H(15)	126.5
N(2)—C(2)—C(1)	111.9 (3)	C(15)—C(16)—H(16)	119.6
N(2)—C(3)—C(4)	105.5 (3)	C(17)—C(16)—H(16)	120.2
N(4)—C(4)—C(3)	106.4 (4)	C(16)—C(17)—H(17)	120.0
N(4)—C(4)—C(5)	123.0 (3)	C(18)—C(17)—H(17)	120.1
C(3)—C(4)—C(5)	130.5 (3)	C(17)—C(18)—H(18)	119.8
C(4)—C(5)—C(6)	122.2 (3)	C(19)—C(18)—H(18)	119.5
C(4)—C(5)—C(10)	120.4 (3)	C(18)—C(19)—H(19)	120.1
C(6)—C(5)—C(10)	117.3 (4)	C(20)—C(19)—H(19)	119.9
C(5)—C(6)—C(7)	121.4 (4)	C(15)—C(20)—H(20)	119.7
C(6)—C(7)—C(8)	120.0 (4)	C(19)—C(20)—H(20)	120.2
C(7)—C(8)—C(9)	119.5 (4)	N(1)—C(21)—H(21)	109.8
C(8)—C(9)—C(10)	120.0 (4)	N(1)—C(21)—H(22)	107.4
C(5)—C(10)—C(9)	121.7 (4)	C(22)—C(21)—H(21)	110.3
N(1)—C(11)—C(12)	113.5 (3)	C(22)—C(21)—H(22)	107.3
N(5)—C(12)—C(11)	113.2 (3)	H(21)—C(21)—H(22)	109.5
N(5)—C(13)—C(14)	106.7 (3)	N(8)—C(22)—H(23)	109.0
N(7)—C(14)—C(13)	106.0 (4)	N(8)—C(22)—H(24)	109.4
N(7)—C(14)—C(15)	122.4 (3)	C(21)—C(22)—H(23)	109.0
C(13)—C(14)—C(15)	131.6 (3)	C(21)—C(22)—H(24)	108.3
C(14)—C(15)—C(16)	119.7 (4)	H(23)—C(22)—H(24)	109.5
C(14)—C(15)—C(20)	121.2 (3)	N(8)—C(23)—H(25)	126.8
C(16)—C(15)—C(20)	119.0 (4)	C(24)—C(23)—H(25)	127.2
C(15)—C(16)—C(17)	120.2 (4)	C(25)—C(26)—H(26)	119.6
C(16)—C(17)—C(18)	119.9 (4)	C(27)—C(26)—H(26)	120.1
C(17)—C(18)—C(19)	120.7 (5)	C(26)—C(27)—H(27)	119.6
C(18)—C(19)—C(20)	120.0 (4)	C(28)—C(27)—H(27)	120.1
C(15)—C(20)—C(19)	120.1 (3)	C(27)—C(28)—H(28)	119.5
N(1)—C(21)—C(22)	112.4 (3)	C(29)—C(28)—H(28)	120.0
N(8)—C(22)—C(21)	111.5 (3)	C(28)—C(29)—H(29)	119.8
N(8)—C(23)—C(24)	106.1 (2)	C(30)—C(29)—H(29)	119.8
N(10)—C(24)—C(23)	107.9 (3)	C(25)—C(30)—H(30)	119.3
N(10)—C(24)—C(25)	122.2 (3)	C(29)—C(30)—H(30)	120.5
			
N(4)—Ag(1)—N(7)^i^—N(6)^i^	104.7 (2)	Ag(1)^xiv^—N(10)—C(24)—C(23)	−170.2 (3)
N(4)—Ag(1)—N(7)^i^—C(14)^i^	−68.5 (3)	Ag(1)^xiv^—N(10)—C(24)—C(25)	8.7 (5)
N(7)^i^—Ag(1)—N(4)—N(3)	96.6 (2)	N(9)—N(10)—C(24)—C(23)	1.4 (3)
N(7)^i^—Ag(1)—N(4)—C(4)	−100.5 (3)	N(9)—N(10)—C(24)—C(25)	−179.6 (3)
N(4)—Ag(1)—N(10)^ii^—N(9)^ii^	66.5 (2)	N(1)—C(1)—C(2)—N(2)	−70.9 (4)
N(4)—Ag(1)—N(10)^ii^—C(24)^ii^	−105.2 (3)	N(2)—C(3)—C(4)—N(4)	0.2 (3)
N(10)^ii^—Ag(1)—N(4)—N(3)	−80.8 (2)	N(2)—C(3)—C(4)—C(5)	−178.9 (3)
N(10)^ii^—Ag(1)—N(4)—C(4)	82.1 (3)	N(4)—C(4)—C(5)—C(6)	20.4 (5)
N(7)^i^—Ag(1)—N(10)^ii^—N(9)^ii^	−111.4 (2)	N(4)—C(4)—C(5)—C(10)	−161.5 (3)
N(7)^i^—Ag(1)—N(10)^ii^—C(24)^ii^	76.9 (4)	C(3)—C(4)—C(5)—C(6)	−160.5 (3)
N(10)^ii^—Ag(1)—N(7)^i^—N(6)^i^	−77.9 (2)	C(3)—C(4)—C(5)—C(10)	17.6 (5)
N(10)^ii^—Ag(1)—N(7)^i^—C(14)^i^	108.9 (3)	C(4)—C(5)—C(6)—C(7)	178.8 (3)
C(1)—N(1)—C(11)—C(12)	162.9 (3)	C(4)—C(5)—C(10)—C(9)	−178.0 (3)
C(11)—N(1)—C(1)—C(2)	−80.9 (4)	C(6)—C(5)—C(10)—C(9)	0.2 (4)
C(1)—N(1)—C(21)—C(22)	−79.6 (4)	C(10)—C(5)—C(6)—C(7)	0.6 (5)
C(21)—N(1)—C(1)—C(2)	158.2 (3)	C(5)—C(6)—C(7)—C(8)	−1.3 (5)
C(11)—N(1)—C(21)—C(22)	159.5 (3)	C(6)—C(7)—C(8)—C(9)	1.1 (5)
C(21)—N(1)—C(11)—C(12)	−76.5 (4)	C(7)—C(8)—C(9)—C(10)	−0.3 (5)
N(3)—N(2)—C(2)—C(1)	−39.0 (4)	C(8)—C(9)—C(10)—C(5)	−0.3 (5)
C(2)—N(2)—N(3)—N(4)	−173.8 (2)	N(1)—C(11)—C(12)—N(5)	−71.1 (4)
N(3)—N(2)—C(3)—C(4)	−0.4 (4)	N(5)—C(13)—C(14)—N(7)	−0.0 (3)
C(3)—N(2)—N(3)—N(4)	0.4 (3)	N(5)—C(13)—C(14)—C(15)	−179.0 (3)
C(2)—N(2)—C(3)—C(4)	173.3 (3)	N(7)—C(14)—C(15)—C(16)	157.3 (3)
C(3)—N(2)—C(2)—C(1)	147.8 (3)	N(7)—C(14)—C(15)—C(20)	−21.3 (5)
N(2)—N(3)—N(4)—Ag(1)	165.6 (2)	C(13)—C(14)—C(15)—C(16)	−23.9 (6)
N(2)—N(3)—N(4)—C(4)	−0.2 (3)	C(13)—C(14)—C(15)—C(20)	157.5 (4)
Ag(1)—N(4)—C(4)—C(3)	−164.4 (2)	C(14)—C(15)—C(16)—C(17)	−180.0 (3)
Ag(1)—N(4)—C(4)—C(5)	14.9 (4)	C(14)—C(15)—C(20)—C(19)	178.9 (3)
N(3)—N(4)—C(4)—C(3)	−0.0 (3)	C(16)—C(15)—C(20)—C(19)	0.2 (5)
N(3)—N(4)—C(4)—C(5)	179.2 (3)	C(20)—C(15)—C(16)—C(17)	−1.3 (6)
N(6)—N(5)—C(12)—C(11)	−117.3 (3)	C(15)—C(16)—C(17)—C(18)	2.1 (6)
C(12)—N(5)—N(6)—N(7)	−177.2 (2)	C(16)—C(17)—C(18)—C(19)	−1.7 (6)
N(6)—N(5)—C(13)—C(14)	−0.1 (3)	C(17)—C(18)—C(19)—C(20)	0.6 (6)
C(13)—N(5)—N(6)—N(7)	0.1 (3)	C(18)—C(19)—C(20)—C(15)	0.2 (5)
C(12)—N(5)—C(13)—C(14)	176.9 (3)	N(1)—C(21)—C(22)—N(8)	−63.9 (3)
C(13)—N(5)—C(12)—C(11)	66.0 (5)	N(8)—C(23)—C(24)—N(10)	−1.4 (4)
N(5)—N(6)—N(7)—Ag(1)^x^	−174.7 (2)	N(8)—C(23)—C(24)—C(25)	179.7 (3)
N(5)—N(6)—N(7)—C(14)	−0.1 (3)	N(10)—C(24)—C(25)—C(26)	−172.5 (3)
Ag(1)^x^—N(7)—C(14)—C(13)	173.7 (2)	N(10)—C(24)—C(25)—C(30)	9.1 (5)
Ag(1)^x^—N(7)—C(14)—C(15)	−7.2 (5)	C(23)—C(24)—C(25)—C(26)	6.2 (6)
N(6)—N(7)—C(14)—C(13)	0.1 (3)	C(23)—C(24)—C(25)—C(30)	−172.2 (4)
N(6)—N(7)—C(14)—C(15)	179.2 (3)	C(24)—C(25)—C(26)—C(27)	−176.0 (3)
N(9)—N(8)—C(22)—C(21)	−49.6 (4)	C(24)—C(25)—C(30)—C(29)	177.9 (4)
C(22)—N(8)—N(9)—N(10)	−178.8 (2)	C(26)—C(25)—C(30)—C(29)	−0.5 (6)
N(9)—N(8)—C(23)—C(24)	0.9 (4)	C(30)—C(25)—C(26)—C(27)	2.4 (5)
C(23)—N(8)—N(9)—N(10)	−0.0 (3)	C(25)—C(26)—C(27)—C(28)	−2.5 (6)
C(22)—N(8)—C(23)—C(24)	179.5 (3)	C(26)—C(27)—C(28)—C(29)	0.7 (6)
C(23)—N(8)—C(22)—C(21)	131.9 (3)	C(27)—C(28)—C(29)—C(30)	1.3 (7)
N(8)—N(9)—N(10)—Ag(1)^xiv^	173.8 (2)	C(28)—C(29)—C(30)—C(25)	−1.3 (7)
N(8)—N(9)—N(10)—C(24)	−0.9 (3)		

Symmetry codes: (i) −*x*+1/2, *y*−1/2, −*z*+1/2; (ii) *x*+1/2, −*y*+1/2, *z*+1/2; (iii) *x*+1/2, −*y*+1/2, *z*−1/2; (iv) *x*+1, *y*, *z*; (v) −*x*+3/2, *y*−1/2, −*z*+1/2; (vi) −*x*+1, −*y*+1, −*z*; (vii) −*x*+1, −*y*+1, −*z*+1; (viii) *x*−1/2, −*y*+1/2, *z*+1/2; (ix) −*x*, −*y*+1, −*z*; (x) −*x*+1/2, *y*+1/2, −*z*+1/2; (xi) *x*−1, *y*, *z*; (xii) −*x*+3/2, *y*+1/2, −*z*+1/2; (xiii) −*x*, −*y*+1, −*z*+1; (xiv) *x*−1/2, −*y*+1/2, *z*−1/2.
